# A Systematic Review of Rehabilitation Interventions for Athletes with Chronic Ankle Instability

**DOI:** 10.3390/jcm15010220

**Published:** 2025-12-27

**Authors:** Marlena Skwiot

**Affiliations:** Faculty of Health Sciences, University of Lomza, ul. Akademicka 14, 18-400 Lomza, Poland; mskwiot@al.edu.pl; Tel.: +48-660-699-238

**Keywords:** CAI, rehabilitation, athletes

## Abstract

**Background:** Ankle sprains affect approximately 8% of the general population, and recurrence occurs in as many as 80% of patients participating in high-risk sports. The aim of this review was to assess the impact of physiotherapy interventions on chronic ankle stability (CAI), providing evidence for the effectiveness of clinical treatment and care for patients with CAI. **Methods:** A systematic review was conducted in accordance with the Preferred Reporting Items for Systematic Reviews and Meta-Analyses (PRISMA) guidelines. Randomized controlled trials (RCTs) evaluating the effectiveness of physiotherapy interventions in athletes with CAI following injury were analyzed. PubMed, Embase, PEDro, and Cochrane electronic databases were searched. A modified McMaster Critical Review Form for quantitative studies was used to assess the methodological quality of the included studies, in accordance with the guidelines. **Results:** The literature search yielded 316 results, of which 13 articles met all required eligibility criteria and were included in the study. The RCTs included 490 athletes with CAI. Interventions included various types of exercises, including balance training (BT), plyometric training, CrossFit, and neuromuscular training. The duration of the intervention was 4–12 weeks. Both subjective and objective measures were used to assess the effectiveness of the therapy in the following seven domains: Dynamic Balance, Static Balance, Patient-Reported Outcomes, Kinematic Outcomes, Proprioception, Body-Composition, and Strength Assessment. **Conclusions:** The evidence supports the effectiveness of rehabilitation interventions in athletes with CAI. Further large-scale randomized controlled trials, incorporating control groups and long-term follow-up, are needed to better determine the robust impact of conservative management on improving both the physical and psychological health of patients with CAI.

## 1. Introduction

Ankle sprain is one of the most common sports injuries among physically active individuals and poses a significant financial burden on the healthcare system [1]. The incidence of sports-related ankle injuries ranges from 25% to 50% [2,3]. Ankle sprains affect approximately 8% of the general population, and up to 80% of patients participating in high-risk sports experience recurrent injuries [4,5].

So far, systematic reviews on CAI have focused on epidemiology [1], postural control [6], or proprioception [7]. The effects of interventions in patients with CAI have also been examined, including BT [8] and passive support of the ankle joint [9]. As a result, there is a need for an updated review of the effectiveness of various interventions in young, active individuals with CAI. This is highly important both for safe participation in sports and from a psychological perspective.

The International Ankle Consortium has defined CAI as a condition characterized by a significant ankle sprain and/or recurrent ankle sprains and/or ankle instability and/or ankle “giving way” occurring at least twice within the past six months [10].

CAI does not only affect the ankle joint but also influences other joints, leading to musculoskeletal problems [11]. Individuals with CAI may exhibit reduced muscle strength and proprioception, as well as a limited range of motion in the joints [12]. As a consequence, compensatory movements involving proximal muscles, such as the hip abductors, may occur in an attempt to maintain balance on the affected limb, resulting in disturbances of central proprioception [13]. There are scientific articles that express the opposite, stating that problems or alterations in tone or aberrant central myotatic reflexes are what produce dysfunctions in biomechanics, and this is what facilitates ankle sprains, not taking into account traumatic sprains [14].

Moreover, patients with CAI exhibit impaired joint position sense during inversion and eversion movements of the ankle joint [7]. CAI may lead to further injuries, such as recurrent ankle sprains, early onset of osteoarthritis, and increased strain on the anterior cruciate ligament. Since CAI can result in numerous negative consequences, it is important to develop preventive strategies to address this ankle joint condition [15].

For patients with CAI, conservative treatment is the first-line approach. If symptoms persist after the completion of conservative therapy, surgical intervention may be considered [16]. This decision should take into account central dysfunction and the elimination of abnormal myotatic reflexes [14].

Various rehabilitation interventions have been examined to address the deficits associated with CAI. These interventions focused on improving progressive strength [17,18] and balance [19,20]. The effectiveness of multicomponent rehabilitation interventions (including strength, balance, and range of motion) has also been investigated, showing that such programs effectively enhance strength, balance, and ankle joint function [21].

Balance training refers to a rehabilitation approach aimed at restoring or improving the body’s balance ability [22]. Previous studies [23,24] have demonstrated the effectiveness of balance training in improving sensory-motor and functional activity in patients with CAI, particularly in terms of function, stability, strength, joint range of motion, and balance. However, other studies [25,26] have shown that balance training is not superior to other conservative treatment methods in terms of self-reported function, ankle strength, balance ability, and range of motion in patients with CAI. It should be taken into account whether the lack of improvement is due to central reflexes [14].

Other interventions for patients with CAI have included manual therapy aimed at improving movement deficits through passive techniques [27]. Ankle joint mobilization—involving rolling, rotation, gliding, and traction—has been shown to enhance and restore physiological joint motion. Previous studies on musculoskeletal disorders [27,28] have demonstrated that manual therapy has a positive effect on recovery and is considered an effective method for improving patient comfort by increasing joint range of motion, stimulating neural pathways, and enhancing proprioception.

It has been shown that in individuals with CAI who do not respond to conservative treatment combined with functional rehabilitation, surgical management may be considered, as it provides positive long-term outcomes [28]. Although numerous surgical techniques have been described, surgical stabilization of CAI can generally be categorized into ligament repair or ligament reconstruction [29].

The aim of this review was to evaluate the impact of rehabilitation interventions on CAI and to provide evidence on the effectiveness of clinical treatment for athletes with CAI.

## 2. Materials and Methods

### 2.1. Design

The systematic review was conducted in accordance with the Preferred Reporting Items for Systematic Reviews and Meta-Analyses (PRISMA) guidelines. The PRISMA 2020 Checklist is attached as Table S1. Only RCTs were eligible for inclusion. Due to the heterogeneity of the included studies, a meta-analysis was not performed; instead, a descriptive synthesis was undertaken. The eligibility criteria based on the Population, Intervention, Comparison, and Outcome (PICO) framework are presented below. 

### 2.2. Eligibility Criteria

Randomized controlled trials (RCTs) evaluating the effectiveness of rehabilitation interventions in athletes with CAI resulting from injury were searched. Studies eligible for inclusion in the review were published between 2017 and 2025.

#### 2.2.1. Population

The study population was required to meet an age criterion of >16 years. The study group had to consist of people who regularly practice sports. No restriction was placed on a single discipline, because researchers often do not specify the sport practiced.

#### 2.2.2. Interventions

Rehabilitation interventions could include various forms such as static and dynamic balance training, proprioceptive training, neuromuscular therapy, and kinesitherapy. Acceptable comparative interventions included control groups (no intervention) or combinations with other training/rehabilitation methods, including manual therapy (but not as a single one-time intervention).

#### 2.2.3. Outcomes

Due to the diversity of rehabilitation intervention outcomes, the search was not limited to specific results. Outcomes of interest included, among others, balance, lower limb functional status, and quality of life. Studies published in languages other than English were excluded at the full-text review stage.

#### 2.2.4. Exclusion Criteria

Exclusion criteria included: CAI resulting from non-sport-related external factors, developmental or neurological causes, prior ankle injury without instability, interventions in the form of bandaging, bracing, or taping, and surgical procedures for CAI. Systematic reviews and meta-analyses were also excluded.

### 2.3. Information Sources

A comprehensive, independent search was conducted by two reviewers across the PubMed, Embase, PEDro, and Cochrane databases for studies published between January 2015 and July 2025. The reference lists of included studies were also examined to ensure all relevant research was identified. In cases of missing data, the study authors were contacted. The search terms included: “Chronic ankle instability AND athletes,” “Physiotherapy,” and “Rehabilitation.”

### 2.4. Selection Process

Titles and abstracts (stage 1) as well as full-text articles (stage 2) were independently screened by two reviewers. Consultation with a third reviewer was not required, as consensus was reached during the screening and selection process.

### 2.5. Data Collection Process and Items

Two reviewers independently extracted the data using a standardized form. The extracted data included sample characteristics, sample size, duration of the rehabilitation process, outcome measurement tools, and intervention characteristics.

### 2.6. Methodological Quality

The included studies were evaluated and categorized according to the “intervention categories” of the National Health and Medical Research Council (NHMRC) hierarchy of evidence. To assess the methodological quality of the included studies, a modified McMaster Critical Review Form for quantitative studies was used, in line with guidelines [30]. This tool evaluated eight main components: the study objective; literature review; study design (all experimental designs); sample (description of participants, sample size justification, ethics and consent); outcomes (reliability and validity, outcome measures used); intervention (description, contamination, and interaction); results (statistical and clinical significance, analysis methods, and dropouts); and conclusions with practice implications (limitations and biases). Each component was rated as “yes,” “no,” “not reported,” or “ND—not applicable.” A score of 1 was assigned for “yes” and 0 for “no” or “not reported”; when the “ND” category was used, the total score was adjusted accordingly. The maximum possible score for a study was 13, depending on the study design and relevant components.

## 3. Results

Table 1 presents a summary of the NHMRC levels of evidence and the scores assigned based on the modified McMaster Critical Review Form for the thirteen eligible studies [31,32,33,34,35,36,37,38,39,40,41,42,43].

### 3.1. Study Inclusion

**Figure 1:**
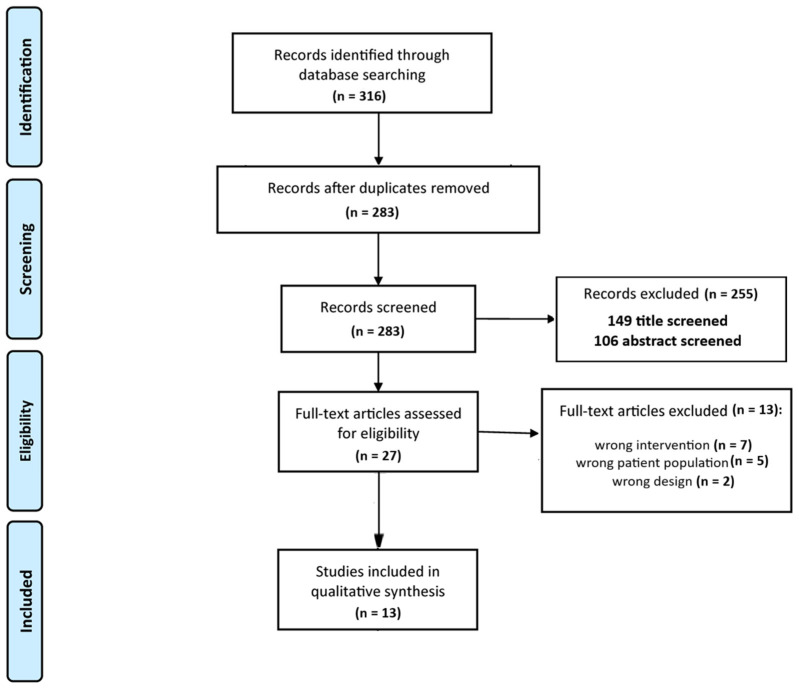
PRISMA 2020 Flow Diagram.

The literature search yielded 316 results, of which 33 duplicates were removed. After screening 283 titles and abstracts, 255 studies were excluded. Full texts of 27 articles were assessed, of which 13 were excluded: 5 for not meeting the study population inclusion criteria, 2 for inappropriate study design, and 7 for unsuitable interventions. 13 articles met all the required eligibility criteria and were included in the review [31,32,33,34,35,36,37,38,39,40,41,42,43]. The PRISMA flow diagram of the study is presented in Figure 1.

#### 3.1.1. Characteristics and Quality of Included RCTs

The characteristics of the included studies are presented in Table 2. The studies originated from the USA (4), Iran (3), Taiwan (2), Korea (1), China (1), Spain (1) and Turkey (1). A total of 490 athletes with CAI participated in the RCTs. The interventions included various types of exercises, such as balance training, plyometric training, CrossFit and neuromuscular training.

Seven studies included a control group with no intervention [32,33,34,35,39,41,43], whereas eleven studies [31,33,34,35,36,37,38,40,41,42,43] included a comparison group that received a different intervention or a combination of interventions. The outcome domains of the studies were categorized into seven groups: static balance, dynamic balance, patient-reported outcomes, kinematics, proprioception, body composition analysis, and strength assessment.

#### 3.1.2. Participants

In the RCT studies, a total of 490 athletes (F = 149; M = 341), aged 16–30 years, participated. All participants were diagnosed with CAI, including ten studies that used the CAIT for assessment. In all cases, the ankle injuries occurred as a result of sports participation, and all athletes received conservative treatment. The participants practiced various sports, including CrossFit [35], Taekwondo [36], and basketball [32]. The remaining studies did not specify the sport disciplines involved.

#### 3.1.3. Intervention Type

Although all studies focused on rehabilitation interventions in patients with CAI, considerable variability was observed both in the types of interventions used and in how they were applied. In three studies [32,39,42], a single intervention was implemented in the experimental group and compared with a control group that received no intervention. In the remaining studies, the effects of different interventions were compared across two or three groups.

##### Balance Training (BT)

In seven RCT [31,32,34,37,38,40,41], BT was used as the intervention; in two studies [33,36], plyometric training was applied. Additionally, individual studies implemented CrossFit training [35], remodeled bicycle pedal training [39], a corrective exercise program [42], and neuromuscular training [43].

In studies utilizing balance training, the primary outcomes assessed were improvements in balance and functional performance. In two studies [31,41], participants underwent stroboscopic training, and the results were compared with those of a control group.

Differences in the duration of interventions between the analyzed studies limit the possibility of directly comparing their results. The varying length of the physiotherapy programs affects the magnitude of the observed effect—shorter interventions may not allow for full patient adaptation, whereas longer ones promote the accumulation of changes. The effectiveness of a 4-week intervention in addressing residual deficits associated with CAI in adolescent patients was confirmed. In two studies, the effectiveness of Hop-Stabilization Training was evaluated in patients [37,42]. A 6-week training program altered landing biomechanics during jumping in basketball players [32] and improved athletes’ performance in the FAAM-ADL, FAAM-Sports, and SEBT tests to a similar extent as traditional stability training. Furthermore, it was shown that both physical exercise and balance training were effective in improving postural control during landing [38], and the addition of vibration led to different improvements in balance ability compared with stability training alone [34]. Balance training and the combination of these two interventions significantly improved static postural stability in athletes with CAI. However, only the intervention methods involving BT were effective in enhancing dynamic stability [40].

##### Plyometric Training and Other Interventions

In two studies [36,43], plyometric training was used to control ankle instability during landing. The CrossFit intervention combined with self-mobilization was superior to the CrossFit training–alone intervention in terms of ankle DFROM, as well as posterolateral and posteromedial reach distances [35]. It was also shown that neuromuscular training (NMT) combined with neurofeedback training (NFT) was more effective than NMT alone in improving postural control during single- and double-leg stance under both eyes-open and eyes-closed conditions, proprioception at 20° of plantar flexion, as well as in reducing anxiety and depression in athletes with CAI [43].

### 3.2. Outcomes Measures

The types of outcome measures used to assess the effectiveness of rehabilitation interventions in athletes with CAI varied across studies. Both subjective and objective measures were employed to evaluate the effectiveness of therapy within the following seven domains: Dynamic Balance, Static Balance, Patient-Reported Outcomes, Kinematic Outcomes, Proprioception, Muscle Activity, and Body Composition.

The studies differed in terms of assessment time points, with outcomes measured over periods ranging from 4 to 12 weeks. No adverse events related to the interventions were reported in any of the studies. The range of domains and outcome measures used in each study is presented in Table 3.

#### 3.2.1. Dynamic Balance

The primary goal of the interventions in the included studies was to improve the functional status of patients with CAI. In 11 out of 13 studies [31,32,33,34,35,36,37,38,39,40,41,42], dynamic balance was assessed. To evaluate the effectiveness of interventions within this domain, various tests were employed, including the SEBT [31,34,35,37,42], Single-Leg Drop Medial Landing Test [33], YBT [36,40], Side-Hop Test [31,38], Figure-8 Hop Test [31], Lateral Shuffling Task [39], and balance assessment platforms [34,38,41].

In all studies utilizing the SEBT, an improvement in balance among athletes was observed following the interventions, which mainly included BT. It should be noted that both the types of interventions and the duration of therapy varied across the studies.

In the studies utilizing the Single-Leg Drop Medial Landing Test, Side-Hop Test, Figure-8 Hop Test, and Lateral Shuffling Task, improvements in balance were observed following the interventions. The studies differed in the type of intervention applied. The intervention duration was similar, ranging from 4 to 6 weeks. Interventions involving Plyometric Training [36] and BT [40] were effective in improving dynamic stability in the YBT. However, combining the program tDCS did not provide any additional benefits.

The platforms used for dynamic balance assessment included the HUBER^®^ 360 (Chattanooga, DJO Global, Guildford, UK) [41], the Biodex Balance System [34], and Bertec Corporation, Columbus, USA [38]. Results obtained with these tools indicated that only exercise-based training and movement rehabilitation produced positive improvements in balance,

#### 3.2.2. Static Balance

The following tools were used to assess static balance outcomes: Time-in-Balance Test, Foot-Lift Test [31], BESS [40], and Center of Pressure [43]. The interventions applied in these studies led to beneficial changes in patients’ balance maintenance strategies. This is evidenced by an increased duration of balance maintenance and a reduction in foot-lifting compensations.

#### 3.2.3. Patient-Reported Outcomes

In this domain, various tools were used to assess overall functioning, as well as to measure anxiety and depression related to CAI.

The FAAM is a valid questionnaire for evaluating functional status in adult patients with CAI [44]. It was used in 6 of the 14 included studies [31,32,37,41,42,43]. The FAAM [45] assesses overall functional status as reported by patients with musculoskeletal injuries and disorders of the lower limbs, ankle, and foot. Additionally, in four studies, the FAAM-S subscale was included. These are common tools for evaluating the effects of balance training. The studies demonstrated the effectiveness of balance training in improving sensorimotor and functional activities in athletes with CAI, including enhancements in function, stability, strength, joint range of motion, and balance.

The CAIT was used in 9 of the 13 RCTs. This tool is designed to assess the severity of functional problems in patients with ankle instability [41]. The CAIT is a valid and reliable questionnaire that can be used to measure the severity of functional difficulties in adults with CAI. In six studies, involving a total of 255 patients, CAIT scores were reported following interventions that included balance, plyometric, vibration, and CrossFit training. All studies observed a significant improvement in those outcomes.

In one RCT [41], the IDFAI was used prior to stroboscopic BT. Scores of 11 or higher allowed for the identification of functional ankle instability in the participating athletes. To assess fear of movement, the TSK was employed [37,38]. This secondary outcome was selected to provide a more holistic evaluation of participants’ functional and psychological well-being [46]. It was shown that after 4 weeks of balance training, fear of movement decreased significantly, as indicated by lower TSK scores following the intervention compared to pre-treatment levels (*p* = 0.027) [38]. In another RCT [43], the HADS [47] was used to assess fear of movement. It is a 14-item scale, with seven items relating to anxiety and seven relating to depression. Neuromuscular training significantly improved participants’ scores on this test.

#### 3.2.4. Kinematics Outcomes

In addition to balance assessment, the researchers analyzed lower-limb kinematic parameters before and after interventions.

In three RCTs, infrared cameras were used to assess kinetics [32,36,39]. However, these studies differed in methodology, the parameters evaluated, and the interventions applied. Ardakani MK et al. [32] used six cameras to capture kinematic values for dorsiflexion, inversion, adduction of the foot, knee flexion, varus alignment, internal rotation, hip flexion adduction, and internal rotation during single-leg landing in basketball players. Similarly, Lee HM et al. [36] analyzed kinematic parameters for hip, knee, and ankle joint angles in the sagittal plane in Taekwondo athletes during jump landing. In contrast, Wu HW et al. [39] measured ankle joint angles during initial contact (IC) and propulsion phases of lateral rolling before and after 6 weeks of modified pedal training.

Another tool used to assess ankle kinematics before and after CrossFit training and CrossFit combined with joint mobilization was DFROM, evaluated using the WBLT [35].

Additionally, Bagherian S. et al. [42] assessed athletes’ movement efficiency using three types of squats: double-limb squat (DLS), double-limb squat with heel lift (DLS-HL), and a squat performed before returning to the starting position, with both descent and ascent phases lasting 2 s.

#### 3.2.5. Proprioception

Previous studies have shown that joint position sense decreases when mechanoreceptors are damaged due to injury [48,49]. Therefore, in studies examining the effectiveness of interventions in athletes with CAI. Some researchers assessed joint position sense (JPS) before and after therapy. Electrogoniometers [33,43], isokinetic dynamometers [42], and a custom experimental device consisting of two inclined boards (the inclined block method, weighted with body mass) [37] were used. The interventions applied produced beneficial effects in improving ankle proprioception, which is essential for proper foot positioning following injury.

#### 3.2.6. Muscles Activity

Additionally, muscle strength was assessed in two RCTs. The studies differed in interventions, methodology, and the muscles evaluated. Using an isokinetic dynamometer, the mean peak torque-to-body-mass ratio was determined for ankle dorsiflexors, plantarflexors, and invertor and evertor muscles [42]. Wu HW et al. [39] used a surface electromyography (sEMG) system to record muscle activity during task performance. The lower-limb muscles examined in athletes with CAI included the tibialis anterior, peroneus longus, gastrocnemius medialis, gastrocnemius lateralis, biceps femoris, semitendinosus, rectus femoris, vastus intermedius, vastus medialis, and vastus lateralis. Improvements in lower-limb muscle activation were observed following all interventions.

#### 3.2.7. Body-Composition Analysis

In one RCT, total and regional body composition was assessed using dual-energy X-ray absorptiometry (DEXA) [34]. No significant changes in body composition variables were observed in athletes after 6 weeks of balance training.

### 3.3. Summary of Results

The outcomes of all 13 studies across the assessed domains are summarized in Table 4. The results suggest that the interventions applied were effective in all included domains. Evidence indicates that physiotherapeutic interventions led to improvements in static and dynamic balance, enhanced proprioceptive sensation, and increased activation of the examined lower limb muscles. Moreover, they contributed to a reduction in fear of movement. Considering the small number of studies, but their high quality, these findings represent promising effects of conservative interventions on functional improvement in athletes with CAI. However, caution is warranted when interpreting the results due to the heterogeneity of the interventions.

### 3.4. NHMRC FORM Framework

Table 5 presents a synthesis of the results using the NHMRC FORM framework. The study results are favourable and encouraging, but the differences in the interventions provided and the small number of studies lowered the overall recommendation.

## 4. Discussion

Impaired postural control in patients with CAI may result from deficits in proprioception or neuromuscular control. Consequently, interventions aimed at improving balance in individuals with CAI have the potential to enhance functional performance of the affected ankle and reduce the risk of reinjury, which may positively impact quality of life and decrease sports-related absenteeism [31].

The primary objective of this systematic review was to evaluate the effectiveness of rehabilitation interventions on CAI in athletes. A moderate evidence base, comprising 13 randomized controlled trials (RCTs), was identified. The included studies demonstrated high methodological quality; however, substantial heterogeneity existed in both the rehabilitation protocols and outcome measures employed. The findings indicate that balance training, plyometric exercises, neuromuscular training (NMT), CrossFit, and modified pedaling interventions may yield beneficial effects across multiple domains in athletes with CAI. Consistent evidence was observed particularly in the improvement of both static and dynamic balance. Additional outcomes explored included kinematics, proprioception, muscle activation patterns, and fear of movement. Despite these encouraging results, interpretation should be cautious due to the limited sample sizes and heterogeneity of the evidence.

The duration of interventions in the reviewed studies ranged from 4 to 12 weeks, which may have influenced the observed outcomes. The heterogeneity of the intervention duration makes it difficult to interpret the results, creating a risk of drawing incorrect conclusions about the effectiveness of the applied procedure. For this reason, differences in outcomes may stem either from the intervention itself or from the length of its application. Within athlete cohorts, effect sizes varied from moderate to large, depending on the intervention duration. Notably, balance training produced improvements in clinical functional measures and patient-reported outcomes after only 4–6 weeks, underscoring the importance of incorporating both objective clinical assessments and patient-reported outcome measures when evaluating rehabilitation progress [31,38]. Postural stabilization programs were effective in enhancing postural control and modifying kinematic patterns in athletes [32,37]. Given that jump landings are common in sports such as basketball and represent a frequent mechanism of ankle sprains, sport-specific jump-landing stabilization programs may reduce the risk of lower limb injuries in this population [32]. Furthermore, athletes engaged in plyometric training adopted modified landing strategies, utilizing knee and hip joints to compensate for ankle instability [36]. Isolated plyometric interventions were shown to facilitate faster activation and stabilization of the plantarflexor muscles during jump landings [33]. Future research should aim to establish optimal exercise protocols or examine combinations of plyometric and other rehabilitative interventions.

Enhancement strategies have also been explored by integrating vibration or stroboscopic vision into balance training [34,41]. The addition of vibration resulted in distinct improvements in balance capabilities compared with balance training alone. Stroboscopic training may provide clinically relevant benefits not only by improving balance outcomes in athletes with CAI but also by enhancing sport-specific rehabilitation phases, through reduced reliance on visual input and improved motor control [41].

Physical characteristics associated with ankle instability include proprioception, postural control, and range of motion [43]. CAI may contribute to the development of negative psychological states, such as depression and anxiety [50]. Effective rehabilitation strategies should therefore consider both neuromuscular and psychosocial components. NMT has been shown to improve biopsychosocial outcomes as measured by the HADS, as well as postural control, ankle proprioception, and overall foot and ankle function [43]. NMT is widely recognized as an effective and evidence-based intervention for enhancing proprioception and reducing the risk of recurrent ankle sprains [51]. Combined protocols integrating NMT with neurofeedback training demonstrated superior efficacy in improving these parameters in athletes with CAI [43]. The Tampa Scale for Kinesiophobia (TSK) has been identified as a reliable tool for assessing fear of movement and anxiety in this population [38].

## 5. Limitations

Although this review was conducted following best practices for systematic reviews (PRISMA), it has several limitations. The search was performed using four electronic databases, which may have resulted in some relevant studies being missed. Another limitation was the exclusion of publications in languages other than English. Ultimately, 13 studies meeting the inclusion criteria were identified, representing a modest body of evidence, albeit with consistent positive findings. The included studies varied in terms of interventions, duration of the intervention and outcome measures; therefore, generalizing these results to the broader population of athletes with CAI should be done with caution. The review was not registered and a protocol was not prepared. The lack of registration is due to time constraints.

## 6. Conclusions

The evidence supports the effectiveness of rehabilitation interventions in athletes with CAI. Further large-scale randomized controlled trials, incorporating control groups and long-term follow-up, are needed to better determine the robust impact of conservative management on improving both the physical and psychological health of patients with CAI.

## 7. Practical Implications

The evidence presented in this review supports the effectiveness of rehabilitation interventions, including balance training, plyometric training, and neuromuscular training, in athletes with CAI. Conservative treatment led to improvements in clinical functional parameters, as well as in patient-reported outcomes, including a reduction in fear of movement. However, due to the limited evidence base, these recommendations should be implemented with caution.

## Figures and Tables

**Table 1:** An overview of the NHMRC levels of evidence and assigned scores based on the Modified McMaster Critical Review Form.

Study	NHMRC Level	Items on Modified McMaster Critical Review Form																Raw Score	
		1	2	3	4a	4b	4c	5a	5b	6a	6b	6c	7a	7b	7c	7d	8	Points	%
Cain [31]	Level II	Y	Y	RCT	43	Y	N/A	Y	Y	Y	NAD	Y	Y	Y	Y	Y	Y	12 out of 13	92%
Ardakani [32]	Level II	Y	Y	RCT	28	Y	Y	Y	Y	Y	NAD	Y	Y	Y	Y	Y	Y	13 out of 13	100%
Huang [33]	Level II	Y	Y	RCT	30	Y	Y	Y	Y	Y	NAD	Y	Y	Y	Y	Y	Y	13 out of 13	100%
Sierra-Guzmán [34]	Level II	Y	Y	RCT	50	Y	Y	Y	Y	Y	NAD	Y	Y	Y	Y	Y	Y	13 out of 13	100%
Cruz-Díaz [35]	Level II	Y	Y	RCT	70	Y	Y	Y	Y	Y	NAD	Y	Y	Y	Y	Y	Y	13 out of 13	100%
Lee [36]	Level II	Y	Y	RCT	14	Y	N	Y	Y	Y	NAD	Y	Y	Y	N	N	Y	10 out of 13	77%
Anguish [37]	Level II	Y	Y	RCT	18	Y	N/A	Y	Y	Y	NAD	Y	Y	Y	Y	Y	Y	12 out of 13	92%
Sepasgozar [38]	Level II	Y	Y	RCT	34	Y	Y	Y	Y	Y	NAD	Y	Y	Y	N	Y	Y	12 out of 13	92%
Wu [39]	Level II	Y	Y	RCT	26	Y	N/A	Y	Y	Y	NAD	Y	Y	Y	Y	Y	Y	12 out of 13	92%
Zhang [40]	Level II	Y	Y	RCT	36	Y	Y	Y	Y	Y	NAD	Y	Y	Y	N	Y	Y	12 out of 13	92%
Uzlaşır [41]	Level II	Y	Y	RCT	39	Y	Y	Y	Y	Y	NAD	Y	Y	Y	N	Y	Y	12 out of 13	92%
Bagherian [42]	Level II	Y	Y	RCT	40	Y	Y	Y	Y	Y	NAD	Y	Y	Y	Y	Y	Y	13 out of 13	100%
Yalfani [43]	Level II	Y	Y	RCT	62	Y	Y	Y	Y	Y	NAD	Y	Y	Y	N	Y	Y	12 out of 13	92%

Y: Yes; N: No; NAD: not addressed; N/A: not applicable; ≥85% High methodological quality; 70–84% Medium/moderate quality; <70% Low quality—high risk of methodological bias.

**Table 2:** The study characteristics.

Study	Sample Size	Mean Age	Gender	Discipline	Injury	Intervention	Comparator/Control	Outcome Addressed	Follow Up
Cain [31]	43	G1: 16.42 ± 1.00; G2: 16.40 ± 0.97; G3: 16.20 ± 1.14; Controll: 16.45 ± 1.04	F: 23; M: 20	N/A	CAI	Stroboscopic balance trening	Combination with resistance-band/no intervention	Time-in-Balance-Test, Foot-Lift-Test, (SEBT), Side-Hoop-Test, Figure-8 Hop Test, FAAM, CAIT	4–6 weeks
Ardakani [32]	28	G1: 22.78 ± 3.09; controll: 22.57 ± 2.76	M: 28	Basketball	CAI	Hop-Stabilization Training	No intervention	FAAM, FAAM-S, CAIT	6 weeks
Huang [33]	30	G1: 23.20 ± 2.82; G2: 23.80 ± 4.13; Controll: 23.50 ± 3.00	F: 23; M: 7	N/A	CAI	Plyometric training,	Plyometric training + balance training/no intervention	Single-leg drop medial landing test, CAIT, JPS	6 weeks
Sierra-Guzmán [34]	50	G1: 22.4 ± 2.6; G2: 21.8 ± 2.1; Controll: 23.6 ± 3.4	F: 17; M: 33	N/A	CAI	Wholebody–vibration (WBV) training	Stabilization training without vibration/no intervention	CAIT, BBS platform, SEBT, Body-Composition Analysis	6 weeks
Cruz-Díaz [35]	70	G1: 29.16 ± 8.38; G2: 27.63 ± 7.42; Controll: 30.38 ± 9.86	F: 30; M: 40	CrossFit	CAI	CrossFit training,	CrossFit plus self-mobilization/no intervention	CAIT, SEBT, WBLT-DFROM	12 weeks
Lee [36]	14	G1: 22.00 ± 1.73; G2: 23.57 ± 1.62	M: 14	Taekwondo	CAI	Plyometric training,	Stability exercises	YBT	8 weeks
Anguish [37]	18	G1: 18.44 ± 1.87; G2: 18.33 ± 1.87	F: 2; M: 16	N/A	CAI	Progressive hop-to-stabilization balance (PHSB)	Single-limb balance (SLB) programme	SEBT, FAAM, FAAM-S, JPS	4 weeks
Sepasgozar [38]	34	G1: 22; Controll: 24	F: 11; M: 23	N/A	CAI	Balance training	Exergaming	CAIT, platform, TSK	4 weeks
Wu [39]	26	G1: 22.6 ± 62.47; G2: 22.86 ± 1.78	F: 8; M: 18	N/A	CAI	Remodeled bicycle pedal training	No intervention	CAIT, Lateral Shuffling Task, kinematic assessment, sEMG	6 weeks
Zhang [40]	36	G1: 20.56 ± 1.67; G2: 20.78 ± 1.48; G3: 20.00 ± 1.00; G4: 19.78 ± 0.97	F: 16; M: 20	N/A	CAI	Transcranial direct current stimulation (tDCS), balance training,	tDCS/balance training	CAIT, BESS, YBT	4 weeks
Uzlaşır [41]	39	G1: 19.08 ± 0.40; G2: 20.46 ± 0.51; Controll: 20.23 ± 0.39	F: 19; M: 20	N/A	CAI	Stroboscopic balance training program	Non-strobe/no intervention	FAAM, FAAM-S, IDFAI	6 weeks
Bagherian [42]	40	G1: 21.2 ± 1.7; Controll: 20.9 ± 1.8	M: 40	N/A	CAI	Corrective exercise program	No intervention	FAAM, FAAM-S, squats, SEBT	8 weeks
Yalfani [43]	62	G1: 22.14 ± 2.53; G2: 21.73 ± 2.60; Controll: 21.45 ± 3.21	M: 62	N/A	CAI	Neuromuscular training	Neurofeedback training + neuromuscular training/no training	JPS, FABQ, HADS, FAAM	8 weeks

BESS—Balance Error Scoring System; CAIT—Cumberland Ankle Instability Tool; DFROM—dorsifelxion ROM; FAAM—Foot and Ankle Ability Measure; FAAM-S—Foot and Ankle Ability Measure Sport; HADS—The Hospital Anxiety and Depression Scale; IDFAI—Identification of Functional Ankle Instability Questionnaire; JPS—joint position sense; tDCS—transcranial direct current stimulation; TSK—Tampa Scale for Kinesiophobia; SEBT—Star Excursion Balance Test; YBT—Y-Balance Test.

**Table 3:** Outcome domain and outcome measures used in each study.

Study	Outcome Domain and Outcome Measures																							
	Static Balance				Dynamic Balance							Patient-Reported Outcomes						Kinematic Outcomes			Proprioception	Muscles Activity		Body-Composition Analysis
	Time-in-Balance	Foot-Lift	BESS	Center of Pressure	SEBT	Single-Leg Drop Medial Landing	YBT	Platform	Side-Hop	Figure-8 Hop	Lateral Shuffling Task	FAAM	FAAM-S	CAIT	IDFAI	TSK	HADS	Cameras Infrared	WBLT-DFROM	Squat	JPS	Isokinetic Dynamometer	sEMG	Dual-Energy X-Ray Absorptiometry
Cain	✓	✓			✓				✓	✓		✓		✓										
Ardakani												✓	✓	✓				✓						
Huang						✓								✓							✓			
Sierra-Guzmán					✓			✓						✓										✓
Cruz-Díaz					✓									✓					✓					
Lee							✓											✓						
Anguish					✓							✓	✓								✓			
Sepasgozar								✓	✓					✓		✓								
Wu											✓			✓				✓					✓	
Zhang			✓				✓							✓										
Uzlaşır								✓				✓	✓		✓									
Bagherian					✓							✓	✓							✓	✓	✓		
Yalfani				✓								✓		✓			✓				✓			

BESS—Balance Error Scoring System; CAIT—Cumberland Ankle Instability Tool; DFROM—dorsifelxion ROM; FAAM—Foot and Ankle Ability Measure; FAAM-S—Foot and Ankle Ability Measure Sport; HADS—The Hospital Anxiety and Depression Scale; IDFAI—Identification of Functional Ankle Instability Questionnaire; JPS—joint position sense; SEBT—Star Excursion Balance Test; YBT—Y-Balance Test; sEMG—surface electromyography.

**Table 4:** The results of all studies in the individual domains.

Study	Effect of Physiotherapy Interventions for the Management of																							
	Static Balance				Dynamic Balance							Patient-Reported Outcomes						Kinematic Outcomes			Proprioception	Muscles Activity		Body-Composition Analysis
	Time-in-Balance	Foot-Lift	BESS	Center of Pressure	SEBT	Single-Leg Drop Medial Landing	YBT	Platform	Side-Hop	Figure-8 Hop	Lateral Shuffling Task	FAAM	FAAM-S	CAIT	IDFAI	TSK	HADS	Cameras Infrared	WBLT-DFROM	Squat	JPS	Isokinetic Dynamometer	sEMG	Dual-Energy X-Ray Absorptiometry
Cain	(+)	(+)			(+)				(+)	(+)		(+)		(+)										
Ardakani												(+)	(+)	(+)				(+)						
Huang						(+)								(+)							(+)			
Sierra-Guzmán					(+)			(+)						(+)										(=)
Cruz-Díaz					(+)									(+)					(+)					
Lee							(+)											(+)						
Anguish					(+)							(+)	(+)								(+)			
Sepasgozar								(+)	(+)					(+)		(+)								
Wu											(+)							(+)					(+)	
Zhang			(+)				(=)																	
Uzlaşır								(+)																
Bagherian					(+)															(+)	(+)	(+)		
Yalfani				(+)													(+)				(+)			

BESS—Balance Error Scoring System; CAIT—Cumberland Ankle Instability Tool; DFROM—dorsifelxion ROM; FAAM—Foot and Ankle Ability Measure; FAAM-S—Foot and Ankle Ability Measure Sport; HADS—The Hospital Anxiety and Depression Scale; IDFAI—Identification of Functional Ankle Instability Questionnaire; JPS—joint position sense; SEBT—Star Excursion Balance Test; YBT—Y-Balance Test; sEMG—surface electromyography. (+) improvement; (=) no changes.

**Table 5:** NHMRS FORM framework.

Component	Grade	Comments
1. Evidence base	A—Excellent Level II studies with a low risk of bias	Quantity: 13 studies Participants: 490 athletes with CAI Level II: 13 studies Level III-2: 0 study Level III-3: 0 study Level IV: 0 study
2. Consistency	B—Good Most studies consistent and inconsistency may be explained	Similar time-point measurements Consistent results Heterogeneous interventions Various outcome measures
3. Clinical impact	B—Good Substantial	Consistent outcome results: particularly improved functionality following interventions. All studies demonstrate statistical significance. Clinical significance should be considered with caution. No adverse events were reported.
4. Generalisability	B—Good Population/s studied in the body of evidence are similar to the target population for the guideline	The study population is similar to the target population. Age ranged from 16 to 30 years. All patients were athletes with CAI who were treated conservatively. The studies were conducted in eight different countries with different healthcare contexts.
5. Grade of recommendations	C—Satisfactory Evidence provides some support for recommendation(s) but care should be taken in its application	These studies had high evidence and were of high methodological quality. Although overall there were positive results, the current evidence base is not homogeneous in terms of interventions delivered, and parameters and results measured for athletes with CAI.

## Data Availability

No new data were created or analyzed in this study.

## Supplementary Materials

The following supporting information can be downloaded at: https://www.mdpi.com/article/10.3390/jcm15010220/s1, Table S1: PRISMA 2020 Checklist.

## Abbreviations

The following abbreviations are used in this manuscript:

CAI	The International Ankle Consortium
PRISMA	Preferred Reporting Items for Systematic Reviews and Meta-Analyses
RCT	Randomized Controll Trial
PICO	Population, Intervention, Comparison, and Outcome
BESS	Balance Error Scoring System
CAIT	Cumberland Ankle Instability Tool
DFROM	dorsifelxion ROM
FAAM	Foot and Ankle Ability Measure
FAAM-S	Foot and Ankle Ability Measure Sport
JPS	joint position sense
TSK	Tampa Scale for Kinesiophobia
SEBT	Star Excursion Balance Test
YBT	Y-Balance Test
BT	balance training
NMT	neuromuscular training combined
NFT	neurofeedback training
tDCS	transcranial direct current stimulation
IDFAI	The Identification of Functional Ankle Instability Questionnaire
HADS	The Hospital Anxiety and Depression Scale
DLS	double-limb squat
DLS-HL	double-limb squat with heel lift
sEMG	surface electromyography
DEXA	dual-energy X-ray absorptiometry
